# Fe biomineralization mirrors individual metabolic activity in a nitrate-dependent Fe(II)-oxidizer

**DOI:** 10.3389/fmicb.2015.00879

**Published:** 2015-09-08

**Authors:** Jennyfer Miot, Laurent Remusat, Elodie Duprat, Adriana Gonzalez, Sylvain Pont, Mélanie Poinsot

**Affiliations:** ^1^Institut de Minéralogie, de Physique des Matériaux et de Cosmochimie, Muséum National d’Histoire Naturelle, Université Pierre et Marie Curie – Sorbonne Universités, CNRS UMR 7590, IRD 206Paris, France; ^2^Département des Collections, Muséum National d’Histoire NaturelleParis, France

**Keywords:** nitrate dependent ferrous oxidation, biomoineralization, NanoSIMS, viability, phenotypic heterogeneity

## Abstract

Microbial biomineralization sometimes leads to periplasmic encrustation, which is predicted to enhance microorganism preservation in the fossil record. Mineral precipitation within the periplasm is, however, thought to induce death, as a result of permeability loss preventing nutrient and waste transit across the cell wall. This hypothesis had, however, never been investigated down to the single cell level. Here, we cultured the nitrate reducing Fe(II) oxidizing bacteria *Acidovorax* sp. strain BoFeN1 that have been previously shown to promote the precipitation of a diversity of Fe minerals (lepidocrocite, goethite, Fe phosphate) encrusting the periplasm. We investigated the connection of Fe biomineralization with carbon assimilation at the single cell level, using a combination of electron microscopy and Nano-Secondary Ion Mass Spectrometry. Our analyses revealed strong individual heterogeneities of Fe biomineralization. Noteworthy, a small proportion of cells remaining free of any precipitate persisted even at advanced stages of biomineralization. Using pulse chase experiments with ^13^C-acetate, we provide evidence of individual phenotypic heterogeneities of carbon assimilation, correlated with the level of Fe biomineralization. Whereas non- and moderately encrusted cells were able to assimilate acetate, higher levels of periplasmic encrustation prevented any carbon incorporation. Carbon assimilation only depended on the level of Fe encrustation and not on the nature of Fe minerals precipitated in the cell wall. Carbon assimilation decreased exponentially with increasing cell-associated Fe content. Persistence of a small proportion of non-mineralized and metabolically active cells might constitute a survival strategy in highly ferruginous environments. Eventually, our results suggest that periplasmic Fe biomineralization may provide a signature of individual metabolic status, which could be looked for in the fossil record and in modern environmental samples.

## Introduction

Microbial biomineralization is a widespread process, responsible for the formation of a diversity of minerals (e.g., Ca and Mg-carbonates, iron and manganese oxides, silica, calcium, and iron phosphates…) that can be found in both modern environments and ancient geological formations. This process leads to the close association of microbial cells and minerals that precipitate at the contact of cells or even intracellularly. Highly mineralized conditions are commonly thought to be deleterious and to prevent microorganism growth or even survival. However, bacterial viability is maintained under some mineralized conditions in the environment, as exemplified by the diversity of bacteria thriving in stromatolitic formations (Dupraz and Visscher, 2005; Goh et al., 2009; Gérard et al., 2013). Bacterial survival has also been observed and monitored under experimental conditions, e.g., in encapsulation silica matrices (Nassif et al., 2004; Blondeau and Coradin, 2012; Eleftheriou et al., 2013; Le Ouay et al., 2013). Microorganisms have developed multiple strategies to deal with minerals that are sometimes products of their own metabolism:

(1) Magnetotactic bacteria and some cyanobacteria localize biomineralization intracellularly (Couradeau et al., 2012; Lefevre and Bazylinski, 2013; Benzerara et al., 2014). This provides a fine control of metal transit, mineral composition and/or crystallinity and offers the possibility to take advantage of specific properties of these biominerals (e.g., magnetic properties).
(2) Biomineralization conditions can promote the production of extracellular polymeric substances (EPS) acting as templates for the precipitation of minerals. This has been shown in stromatolites (Decho et al., 2005) and in other systems, among which Fe cycling bacteria. For instance, EPS are produced by the Fe(III)-reducer *Shewanella oneidensis* promoting the precipitation of uraninite (Shao et al., 2014) and by nitrate-dependent iron(II)-oxidizing bacteria precipitating Fe-oxyhydroxides or phosphates (Miot et al., 2009b; Klueglein et al., 2014).
(3) Micro-aerobic iron oxidizing bacteria also produce extracellular organics in the form of stalks (*Gallionella* and *Mariprofundus* genus) or sheaths (e.g., *Leptothrix* or *Sphaerotilus* genus) templating Fe-(oxyhydr)oxide precipitation (Banfield, 2000; Chan et al., 2011, 2009; Seder-Colomina et al., 2014).
(4) In addition, the microaerobic iron-oxidizer *Mariprofundus ferroxydans* PV-1 was shown to exhibit specific cell surface properties mitigating interaction with Fe minerals (Saini and Chan, 2013).
(5) Recently, *S. oneidensis* was shown to form membrane vesicles that get mineralized when exposed to uranium, hence lessening cell surface mineralization (Shao et al., 2014).
(6) Finally, phototrophic iron oxidizing bacteria produce a locally slightly acidic microenvironment around them that could increase Fe(III) solubility in the immediate vicinity of the cells and thus inhibit its precipitation at direct contact of the cells (Hegler et al., 2010).

Nonetheless, there have been numerous reports of microorganisms becoming encrusted within their cell wall in laboratory experiments, modern environments as well as the fossil record. Such periplasmic encrustation has been observed with bacteria mineralized by calcium phosphate (Benzerara et al., 2004; Cosmidis et al., 2013), chromium phosphate (Goulhen et al., 2006), uranyl phosphate (Dunham-Cheatham et al., 2011), As-Fe-hydroxysulfate (Benzerara et al., 2008), Fe sulfide (Donald and Southam, 1999), and a diversity of Fe-minerals (Miot et al., 2011, 2014a,b; Cosmidis et al., 2014; Klueglein et al., 2014). Periplasmic mineralization is a potential consequence of the activity of periplasmic enzymes, promoting (most plausibly indirectly) the precipitation of insoluble elements. For instance, activity of the periplasmic Nar enzyme involved in nitrate reduction was proposed to be responsible for Fe(III) precipitation in the periplasm of some anaerobic iron oxidizing bacteria (Miot et al., 2011; Picardal, 2012; Carlson et al., 2013; Etique et al., 2014; Klueglein et al., 2014). Another example is the precipitation of periplasmic and/or cell surface meta-autunite (uranium phosphate) linked to the expression of a periplasmic alkaline phosphatase and subsequent export of inorganic phosphate in cultures of *Caulobacter crescentus* exposed to uranium (Yung and Jiao, 2014).

However, the periplasm is a key transit site, across which nutrients and wastes circulate. Its encrustation is thus thought to be lethal and/or at least to strongly limit cell – medium exchanges and hence microbial growth. Such hypotheses related to the viability of mineralized bacteria have, however, never been explored down to the single cell level.

In the present study, we evaluated the link between Fe biomineralization and viability of the anaerobic nitrate-dependent Fe(II)-oxidizer *Acidovorax* sp. strain BoFeN1 under biomineralization conditions in batch cultures. We followed these processes down to the single cell level using a combination of electron microscopy and Nano-Secondary Ion Mass Spectrometry (NanoSIMS). This strain has been shown to promote the biomineralization of a diversity of Fe-minerals depending on biomineralization conditions: Fe-phosphates (Miot et al., 2009b), green rust (Pantke et al., 2012), goethite (Pantke et al., 2012), and lepidocrocite (Miot et al., 2014b) sometimes in association with extracellular magnetite (Miot et al., 2014a). In each case, Fe minerals (except extracellular magnetite) were shown to precipitate at least partly within the periplasm and at the cell surface at more advanced stages. We explored how cells assimilated a labeled organic carbon substrate (^13^C-acetate) as a function of the stage of biomineralization and of the nature of the biominerals precipitated in the periplasm. NanoSIMS has been widely used in the last years to investigate single cell metabolism in laboratory and environmental samples, shedding light for instance on strong individual heterogeneities of carbon or nitrogen assimilation (e.g., Musat et al., 2008; Remusat et al., 2012; Zimmermann et al., 2015). In the present study, NanoSIMS analyses provided a way to analyze single cells and discriminate the C assimilation behavior of mineralized vs. non-mineralized bacteria. We show that BoFeN1 community remains viable under biomineralization conditions as a consequence of heterogeneous biomineralization of the cells. In addition, we show that carbon assimilation is correlated with the amount of Fe precipitated at the cell contact.

## Materials and Methods

### Culture and Biomineralization Conditions

All solutions were prepared with sterile milli-Q water degassed under Ar and manipulations were performed in an anaerobic chamber under N_2_ or Ar atmosphere [p(O_2_) < 30 ppm].

The nitrate-reducing Fe(II)-oxidizing bacteria *Acidovorax* sp. strain BoFeN1 was first pre-cultured in an anoxic rich freshwater mineral medium prepared after (Ehrenreich and Widdel, 1994), as previously described (Miot et al., 2014b). This pre-culture medium contained 1 mM phosphate (KH_2_PO_4_), 10 mM nitrate (NaNO_3_), and 5 mM acetate (Na-acetate). Once in the stationary phase (optical density at 600 nm around 0.4), cells were rinsed and transferred to a biomineralization medium. Four different biomineralization media were prepared: Lp-, Mt-, FeP-, and Gt-media. These media have been previously shown to promote the precipitation of lepidocrocite [Lp, (Miot et al., 2014b)], a mixture of lepidocrocite and magnetite [Mt, (Miot et al., 2014a)], Fe-phosphate [FeP, (Miot et al., 2009a)], and goethite [Gt, (Pantke et al., 2012)] respectively. Lp- and Mt-media were composed of NaCl (11.4 mM), Na-acetate (5 mM), and FeCl_2_ (10 mM), supplemented with vitamins, trace elements and selenite solutions prepared after (Ehrenreich and Widdel, 1994). These media did not contain phosphate and thus were not aimed at promoting bacterial growth. Their pHs were adjusted with NaOH at 7.0 (Lp-medium) or 7.6 (Mt-medium). NaNO_3_ (10 mM) was then added leading to the precipitation of green rust (with a much smaller amount in the Lp-medium; Miot et al., 2014a,b). Cells were transferred in Lp- and Mt-media at 50% (v/v). The FeP-medium was composed of 1 mM phosphate provided as KH_2_PO_4_ and 5 mM Fe(II) provided as FeCl_2_, complemented with vitamins, trace elements and selenite solutions, as well as 5 mM Na-acetate and 10 mM NaNO_3_. A white precipitate mostly composed of vivianite [Fe^II^_3_(PO_4_)_2_⋅8H_2_O] formed, that was removed by filtration at 0.2 μm to obtain the Gt-medium. Bacteria were inoculated in the FeP and Gt media at 10% (v/v).

Bacteria were sampled from the biomineralization media at different stages of biomineralization (t_min_ = 4 h, 1 day, 4 days). They were harvested by centrifugation (6 000 *g*, 15 min) and rinsed twice before being transferred to a labeled medium at a dilution of 10% (v/v). The labeled medium had the same composition as the pre-culture medium but contained a mixture of labeled and non-labeled sources of carbon, namely ^13^C-acetate (40% vol) and ^12^C-acetate (60% vol). This medium was thus nutrient-rich but did not contain iron and thus could not promote biomineralization. Cells grown in the labeled medium were sampled at successive stages of growth (t_lab_ = 4 h, 1 day, 4 days).

### NanoSIMS

Bacteria sampled from the labeled medium and rinsed with degassed milli-Q water were filtered through 0.2 μm polycarbonate filters previously gold coated (20 nm thickness). Quantitative ion images were acquired on the NanoSIMS 50 (Cameca, Gennevilliers, France) installed at the Museum National d’Histoire Naturelle of Paris, France. A 0.8 pA Cs^+^ primary beam was rastered over a surface area of 40 μm × 40 μm, divided into 512 pixels × 512 pixels, at 1 ms/pixel raster speed. Secondary ion images of ^16^O^-^, ^12^C_2_^-^, ^12^C^14^N^-^, ^13^C^14^N^-^, and ^31^P^16^O^-^ were acquired in multicollection mode. The mass resolving power was adjusted at 9000 to resolve isobaric interferences such as ^12^C^14^N^-^ from ^12^C_2_H_2_^-^ or ^13^C^14^N^-^ from ^12^C^15^N^-^. Prior to analysis the sample surface was presputtered using a 80 pA Cs^+^ beam for 5 min over 50 μm × 50 μm to remove surface contamination and reach sputtering steady state. Images were processed using the L’IMAGE software (L.Nittler, Carnegie Institution, Washington, DC, USA) and corrected for detector dead time (44 ns, set electronically) and drift in X and Y positions. A type 3 kerogen standard was used to check for instrumental stability over the course of the analytical session. We chose to map the ^13^C^14^N^-^/^12^C^14^N^-^ ratio (hereafter named ^13^C^14^N/^12^C^14^N) to track the ^13^C isotopic label because the CN^-^ signal is dominated by bacteria contribution; on the other hand, C^-^ signal is overwhelmed by the polycarbonate filter contribution. Regions of interest (ROI) were manually drawn around individual cells in order to calculate their isotopic and elemental compositions. The elemental ratio ^16^O^-^/^12^C^14^N^-^ (hereafter ^16^O/^12^C^14^N) was used to identify extracellular minerals and mineralized cells.

### Statistical Analyses

Levels of carbon assimilation by cell subpopulations or samples were analyzed by statistical comparisons of mean ^13^C^14^N/^12^C^14^N ratios (multiple and pairwise comparisons by one-way ANOVA and Welch two-sample *t-*tests with Bonferroni correction for multiple testing, respectively). The cell dataset used for statistical analyses is composed only of ROI located outside Fe aggregates (Supplementary Table S1). Corrected *p*-values smaller than 0.05 were considered significant.

Parameter estimation of an exponential model was achieved by non-linear least-squares fitting; the goodness of fit was evaluated by *R*-squared value, calculated as 1 minus the ratio of the residual sum of squares to the total sum of squares.

### X-Ray Diffraction

Mineralogy of the products of Fe(II) oxidation was checked using X-ray Diffraction (XRD). Independent cultures were grown in the different media for 4 days. Solid phases were subsequently collected by centrifugation (5500 *g*, 15 min), rinsed twice with degassed milli-Q water and vacuum dried. Analyses were performed within an anoxic cell equipped with a Kapton window and mounted in a glovebox under Ar atmosphere. XRD measurements were performed with Co Kα radiation on a Panalytical X’Pert Pro MPD diffractometer equipped with an X’celerator© detector mounted in Bragg-Bertano configuration. A continuous collection mode was applied over the 10°–90° 2θ range with a 0.033° 2θ step and a total counting time of around 1 h 30 min.

### Scanning Electron Microscopy

Areas on filters analyzed by NanoSIMS were subsequently analyzed by scanning electron microscopy (SEM). SEM observations were performed with a TESCAN VEGA II LSU microscope working with a tungsten filament gun and a ZEISS Ultra 55 SEM equipped with a field emission gun (FEG) in backscattered electron mode at 10 or 15 kV and a working distance of 7.5–15.4 mm. Energy Dispersive X-ray spectrometry analyses were conducted and Fe elemental maps obtained on the same areas using a silicon drift detector at an accelerating voltage of 15 kV and a working distance of 15.4 mm.

### Transmission Electron Microscopy

Independent experiments were conducted for TEM analyses. Cells collected from the biomineralization media were harvested by centrifugation and rinsed twice with degassed milli-Q water. Whole cells were deposited on carbon-coated 200-mesh copper grids.

TEM observations were performed with a FEG JEOL2100F and a LaB6 JEOL2100 microscopes operating at 200 kV. Selected-area diffraction (SAED) patterns were obtained on areas of interest to characterize amorphous vs. (nano) crystalline mineral phases.

## Results

### Co-Existence of Mineralized and Non-Mineralized Cells Under Biomineralization Conditions

**FIGURE 1:**
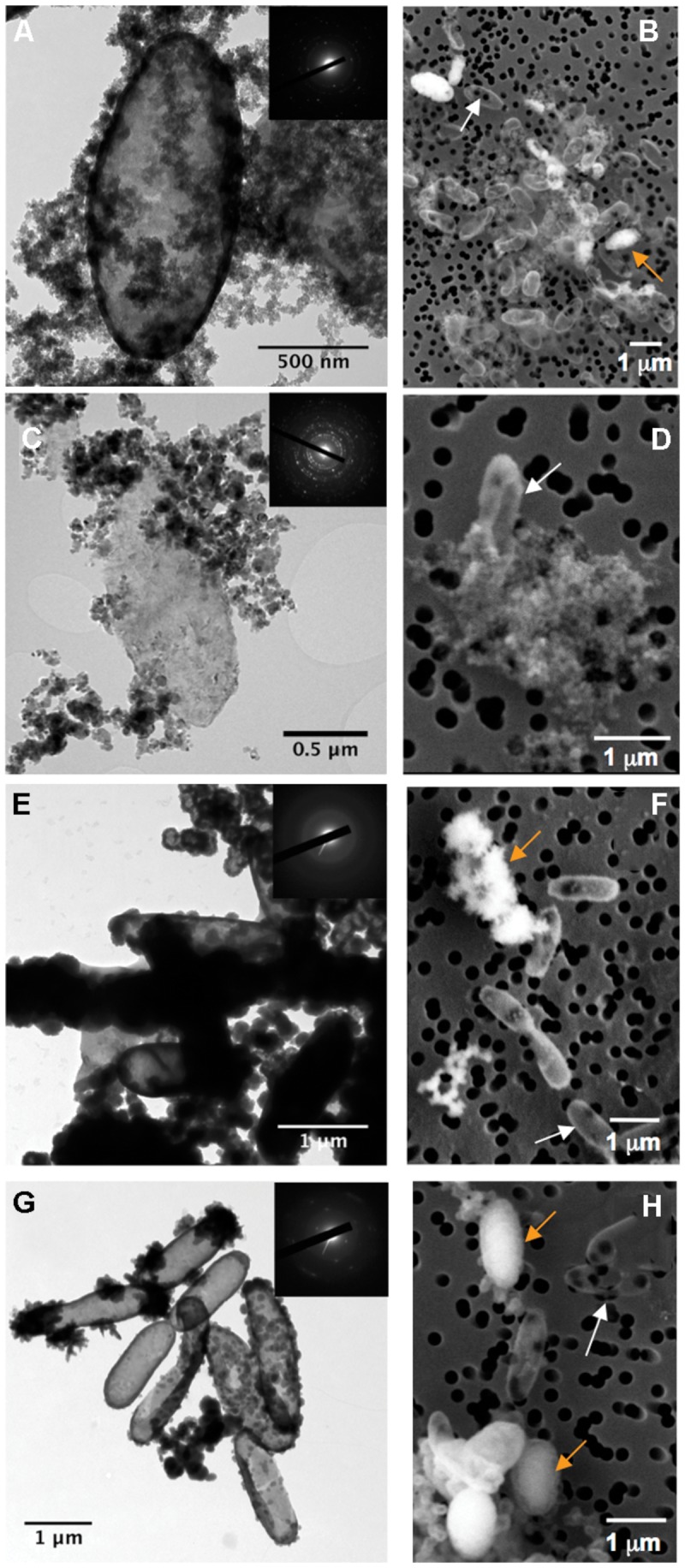
**Electron microscopy analysis of BoFeN1 cells cultured in the different biomineralization media: **(A,B);** Lp-, **(C,D);** Mt-, **(E,F);** FeP- and **(G,H);** Gt-media. (A,C,E,G)** are TEM images and **(B,D,F,H)** are scanning electron microscopy (SEM) images in secondary electron mode. Insets display corresponding selected area electron diffraction (SAED) patterns of lepidocrocite **(A)**, magnetite **(C)**, amorphous Fe-phosphate **(E)**, and goethite **(G)**. White and orange arrows indicate non-encrusted vs. encrusted cells, respectively.

In biomineralization media, BoFeN1 cells get encrusted with Fe-minerals. As previously described, different minerals are produced depending on the medium composition and the nature of Fe source (Kappler et al., 2005; Miot et al., 2009a,b, 2014a,b; Pantke et al., 2012). In a pH neutral medium without phosphate (Lp-medium), lepidocrocite is precipitated in the periplasm as well as in the extracellular medium [**Figure 1A**, (Miot et al., 2014b)]. At pH 8, green rust precipitates as long as nitrate is added to the medium. Bacteria further transform green rust and dissolved Fe(II) into extracellular magnetite and periplasmic lepidocrocite [Mt-medium, **Figure 1C**, (Miot et al., 2014a)]. In the presence of 1 mM phosphate and 5 mM Fe (FeP-medium), amorphous Fe phosphate is precipitated in the periplasm as well as at the cell surface [**Figure 1E**, (Miot et al., 2009a)]. At lower phosphate concentrations (<1 mM; Gt-medium), goethite precipitates both in the periplasm and at the cell contact [**Figure 1G**, (Pantke et al., 2012)]. Hence, whatever the composition of the biomineralization medium, periplasmic precipitation of Fe-minerals is observed, whereas extracellular precipitation of Fe-bearing phases is more or less extensive depending on the chemical conditions. After biomineralization, cells have been transferred to a labeled medium. This labeled medium did not contain iron thus could not promote any further biomineralization.

Whatever the stage and conditions of biomineralization, we observed strong heterogeneities of biomineralization from one cell to another (**Figures 1B,D,F,H**). Indeed, non-mineralized, partially mineralized and completely mineralized cells co-existed in the cultures, even at advanced stages of mineralization (t_min_ = 4 days). These heterogeneities were further investigated by NanoSIMS.

**FIGURE 2:**
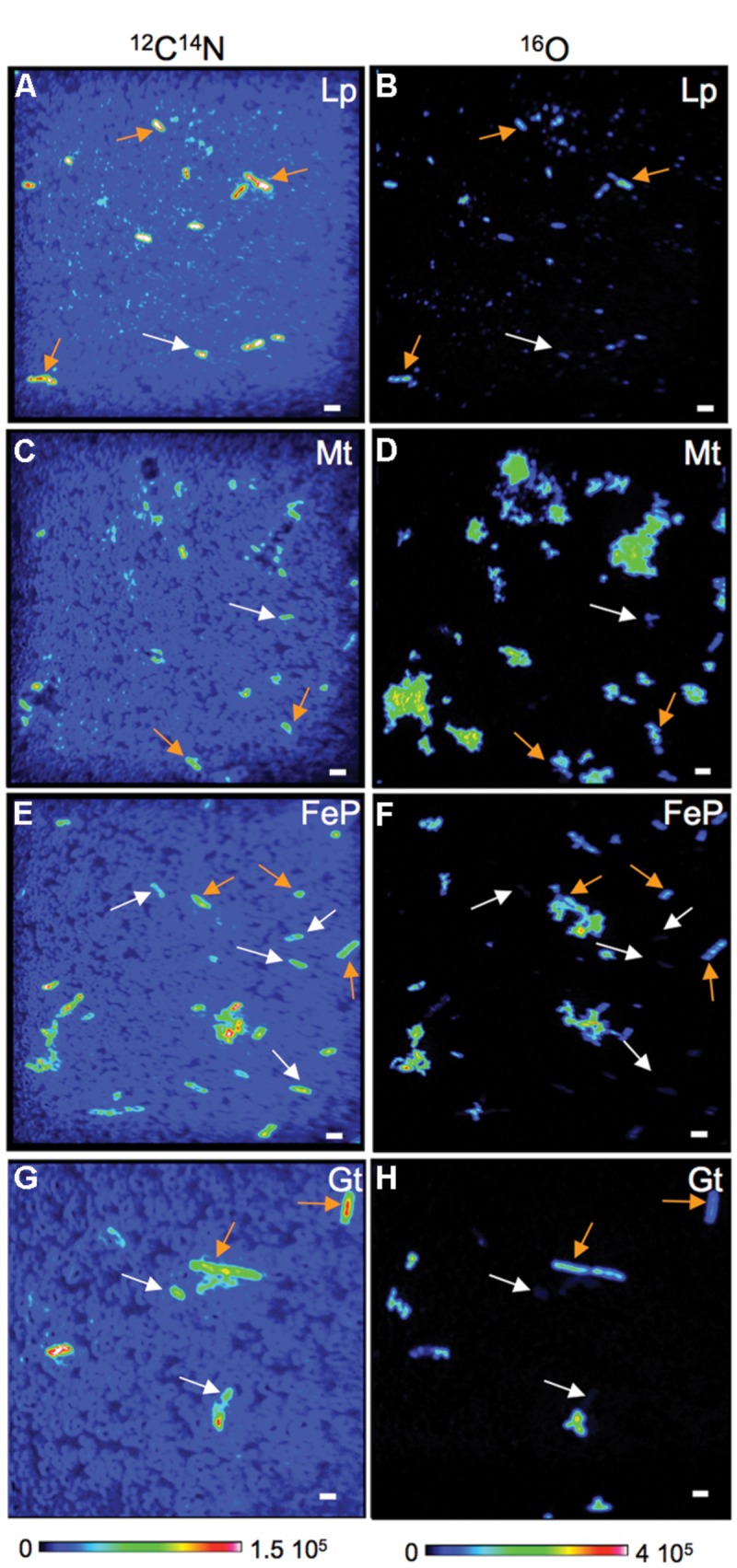
**Co-existence of mineralized and non-mineralized cells in cultures of BoFeN1 collected from biomineralization media. (A–H):** NanoSIMS images (^12^C^14^N and ^16^O) of BoFeN1 grown in biomineralization medium (Lp-, Mt-, FeP-, or Gt-media) for 4 days (t_min_ = 4 days) then transferred to labeled medium for 4 h (t_lab_ = 4 h). Scale bars: 2 μm.

**FIGURE 3:**
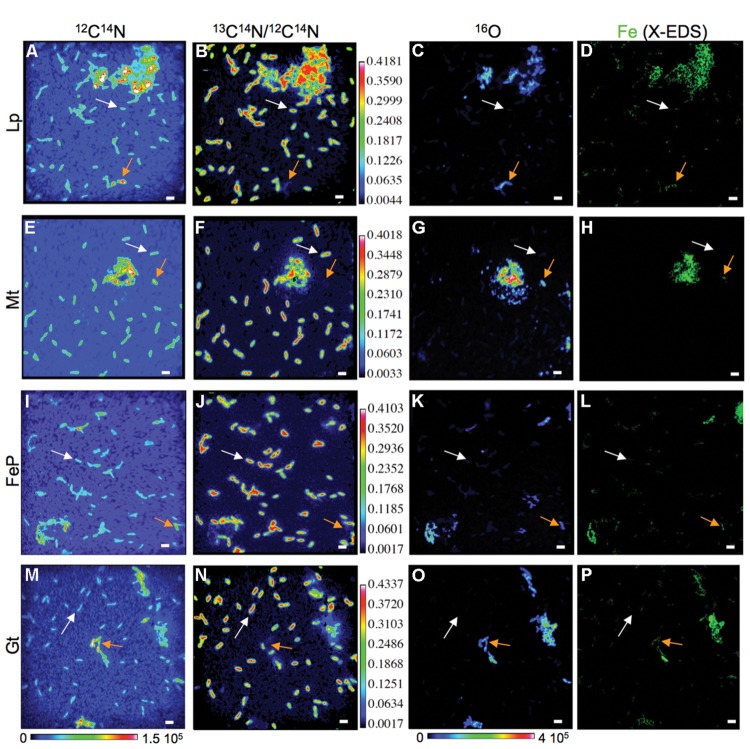
**Differences in carbon assimilation among mineralized (orange arrows) and non-mineralized (white arrows) cells in cultures of BoFeN1 grown for 4 days (t_min_ = 4 days) in biomineralization medium (Lp-, Mt-, FeP-, or Gt-media) then transferred to labeled medium for 4 days.** NanoSIMS images of ^12^C^14^N^-^ signal **(A,E,I,M)**, ^13^C^14^N/^12^C^14^N ratio **(B,F,J,N)** and ^16^O^-^ signal **(C,G,K,O)** and corresponding X-EDS maps showing the distribution of Fe (green, **D,H,L,P**). Scale bars: 2 μm.

**Figure 2** displays NanoSIMS images obtained on cells collected after 4 days of biomineralization that spent only 4 h in the labeled medium. In order to differentiate mineralized vs. non-mineralized cells, we mapped both ^12^C^14^N^-^ and ^16^O^-^ secondary ions. On the one hand, the ^12^C^14^N^-^ signal gives the distribution of organic matter (mainly bacteria) in the samples. On the other hand, we used ^16^O^-^ as a proxy to locate Fe-minerals. Comparison of Fe EDX maps collected in the SEM and ^16^O^-^ maps collected by NanoSIMS on the same areas (**Figure 3**) confirm the relevance of this proxy for Fe-mineral location (though not for Fe quantification). For simplicity, these signals are hereafter labeled ^12^C^14^N and ^16^O. As observed in **Figure 2**, most cells that can be seen on the ^12^C^14^N maps also show up in the corresponding ^16^O maps. These cells are thus mineralized. However, a few cells in the ^12^C^14^N map are not or only poorly visible in the ^16^O map (white arrows in **Figure 2**), indicating that a non-negligible proportion of cells are not mineralized, even after 4 days in the biomineralization medium. The co-existence of mineralized and non-mineralized cells is observed in all four media, i.e., whatever the biomineralization conditions. However, it should be noted that discrimination of mineralized vs. non-mineralized cells is more difficult in samples from Lp- and Mt-media, where extracellular precipitates are more abundant and tend to form aggregates (**Figure 1**). As a consequence, we took into account only cells outside Fe aggregates for the following analyses. As we do not know whether mineralized vs. non-mineralized cells have different behaviors regarding aggregation, it is possible that we under-estimated one of these two groups of cells in the Lp- and Mt-samples.

**FIGURE 4:**
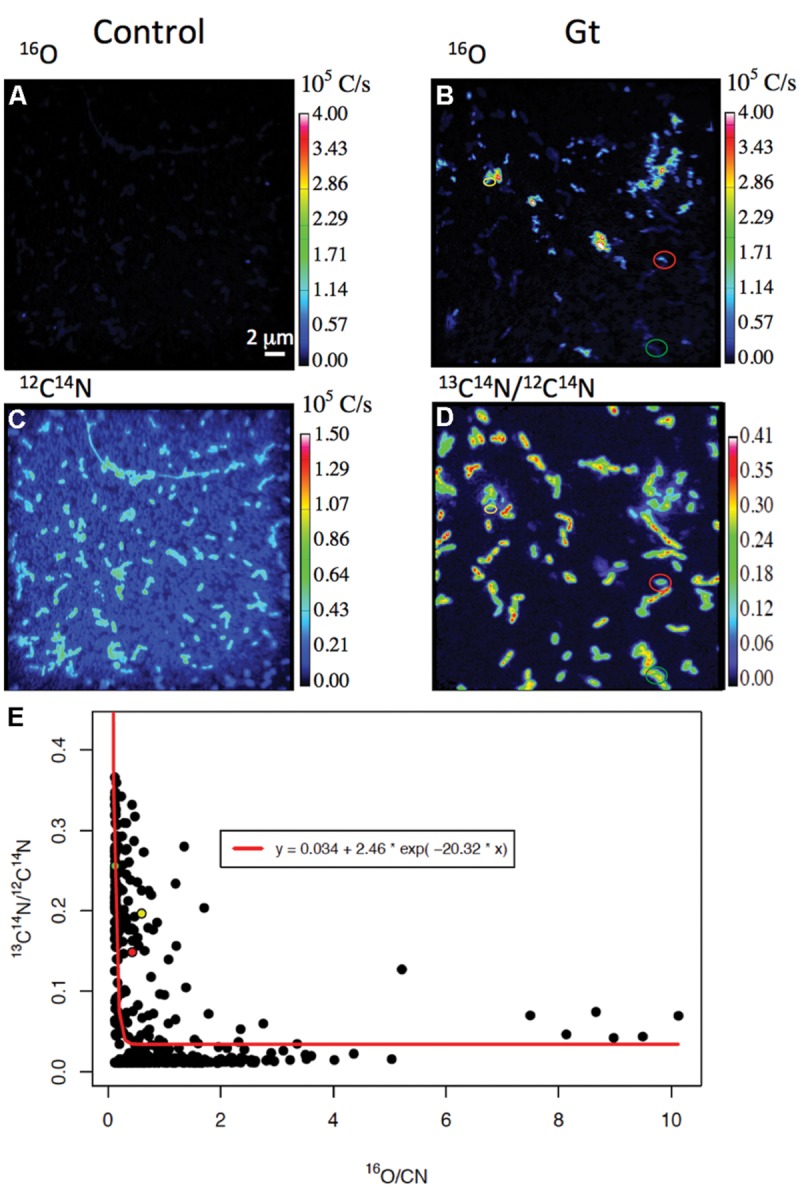
**^13^C assimilation as a function of the level of biomineralization. (A,B)** ^16^O and ^12^C^14^N NanoSIMS images of a control culture never exposed to biomineralization medium. **(C–E)** Carbon assimilation by mineralized cells. **(C,D)** ^16^O and ^13^C^14^N/^12^C^14^N NanoSIMS images of bacteria cultured 1 day in the Gt-medium, then transferred for 1 day in the labeled medium (same area analyzed in the two maps). Red, green, and yellow data points in panel **(E)** correspond to the bacteria identified by the color circles in panels **(C,D)**. Their ^16^O/CN and ^13^C^14^N/^12^C^14^N ratios are (0.429; 0.148), (0.129; 0.255), and (0.598; 0.196), respectively. **(E)** ^13^C^14^N/^12^C^14^N as a function of ^16^O/CN ratios in all mineralized populations of all samples studied (whatever the biomineralization and incubation conditions – see red points in **Figure 6**). The 3-parameter exponential model, estimated by non-linear least-square fitting, is displayed as a red curve (*R*^2^ = 0.397).

**FIGURE 5:**
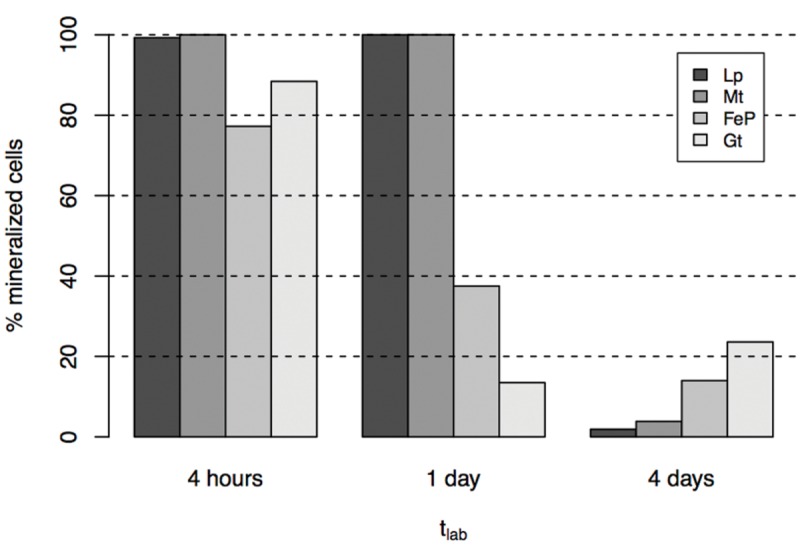
**Proportion of mineralized cells in cultures grown in biomineralization medium for 4 days (t_min_ = 4 days) then transferred to labeled medium for 4 h to 4 days**.

Nano-Secondary Ion Mass Spectrometry analyses indicate that co-existence of mineralized and non-mineralized cells persisted after 4 days in the labeled medium, whatever the biomineralization conditions previously experienced (**Figures 3** and **4**). Based on the maximum value of ^16^O/CN (where CN = ^12^C^14^N + ^13^C^14^N) recorded on a control culture that was prepared without Fe in an unlabeled medium (**Figures 4A,B**), we defined a ^16^O/CN ratio threshold (^16^O/CN = 0.121) above which cells were considered mineralized and below which they were defined as non-mineralized. In the following, quantitative analyses are based on this ^16^O/CN threshold. Cells at early stages of biomineralization exhibit a ^16^O level much higher than cells from the control culture (see e.g., cells circled in red, green, and yellow in **Figures 4C,D**). We estimated the proportion of mineralized vs. non-mineralized cells based on the number of ROIs that exhibited ^16^O/CN ratios above and below the threshold, respectively (Supplementary Table S1, **Figure 5**). The values obtained must be interpreted cautiously as they were not compared with bulk measurements. General trends can, however, be described. The proportion of mineralized cells decreased with increasing time spent in the labeled medium. Significant differences were, however, observed depending on the biomineralization conditions. Proportion of mineralized cells decreased from 77 to 37% and from 88 to 13% in samples from FeP- and Gt-media, respectively, after only 1 day in the labeled medium. By comparison, proportion of mineralized cells remained constant (around 100%) in samples from Lp- and Mt-media at t_lab_ = 1 day. In these samples, the main drop in the number of mineralized cells occurred between t_lab_ = 1 and 4 days. After 4 days in the labeled medium, whatever the biomineralization conditions, all samples were mainly composed of non-mineralized cells (around or less than 20% of mineralized cells remaining).

### Carbon Assimilation by Mineralized Cells

We followed ^13^C-acetate incorporation by cells previously exposed to biomineralization conditions at the single cell level using NanoSIMS maps of the ^13^C^14^N/^12^C^14^N ratio. **Figure 3** displays typical ^13^C^14^N/^12^C^14^N maps obtained for samples exposed for 4 days to biomineralization and then cultured for 4 days in the labeled medium. Non-mineralized cells (identified using the combination of ^12^C^14^N and ^16^O maps) are clearly visible on the corresponding ^13^C^14^N/^12^C^14^N maps, indicating that they significantly incorporated acetate from the labeled medium (white arrows). On the contrary, mineralized cells (orange arrows) are absent from the ^13^C^14^N/^12^C^14^N maps, suggesting that they did not incorporate acetate.

**FIGURE 6:**
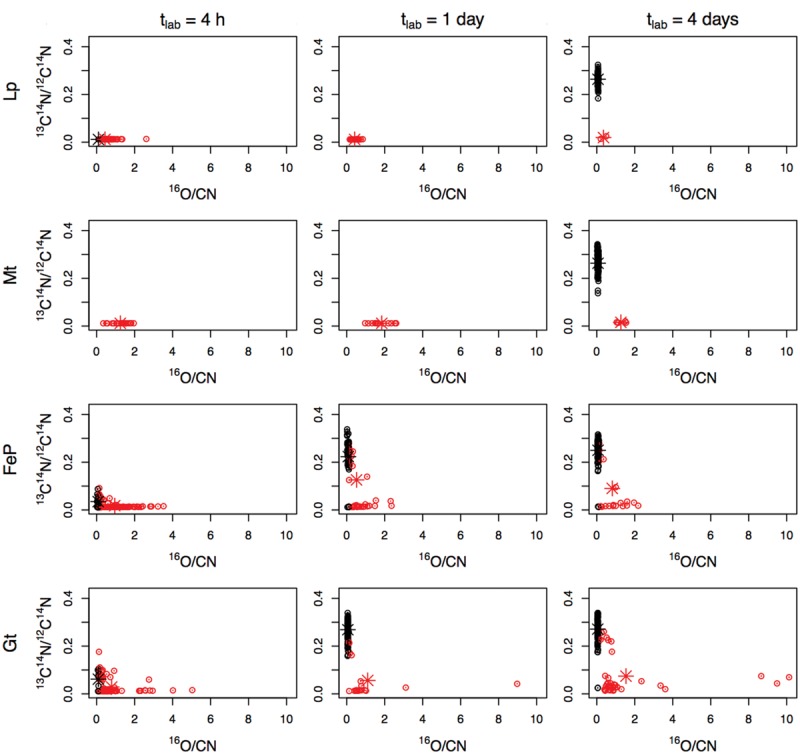
**^13^C^14^N/^12^C^14^N as a function of ^16^O/CN determined by NanoSIMS in single mineralized (red) and non-mineralized (black) cells grown for 4 days (t_min_ = 4 days) in biomineralization medium (Lp-, Mt-, FeP-, Gt-media) then transferred for 4 h, 1 day, or 4 days in labeled medium.** Errors on y-axis values were calculated as (1/^13^C^14^N + 1/^12^C^14^N)^1/2^, and correspond to negligible values; the representation of data (circles) is larger than and encompasses the error bars. The barycenter of each group of points (mineralized vs. non-mineralized subpopulations) is displayed as (red vs. black) star.

**FIGURE 7:**
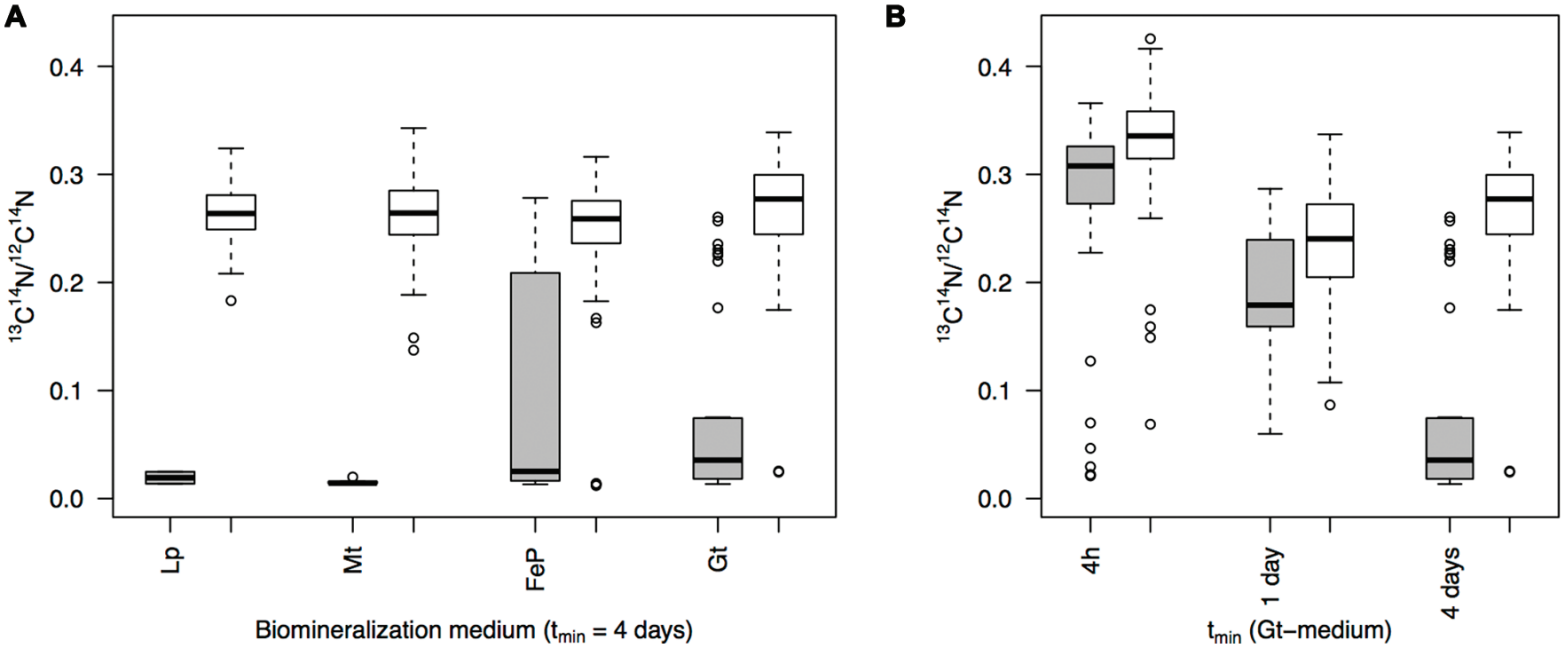
**(A)** ^13^C^14^N/^12^C^14^N ratio distributions as deduced from NanoSIMS analyses in mineralized (gray) vs. non-mineralized (white) populations from cultures grown for 4 days (t_min_ = 4 days) in different biomineralization media (Lp-, Mt-, FeP-, Gt-) then transferred to labeled medium for 4 days (t_lab_ = 4 days). **(B)** Evolution of ^13^C^14^N/^12^C^14^N ratio distributions with increasing time of mineralization (Gt-medium), determined by NanoSIMS analyses of single mineralized (gray) vs. non-mineralized (white) cells in cultures incubated for 4 days in the labeled medium (t_lab_ = 4 days). **(A,B)** For a given distribution, the box boundaries indicate the first and third quartiles; the bold band inside the box corresponds to the median value. The vertical lines that extend from the box encompass the largest/smallest observation that falls within a distance of 1.5 times the box size from the nearest box hinge. Outliers are shown separately (individual points).

This trend is confirmed by the plots displaying ^13^C^14^N/^12^C^14^N as a function of ^16^O/CN in the different samples that were exposed for 4 days to biomineralization conditions before being transferred to the labeled medium (for 4 h, 1 day or 4 days; **Figure 6**). Within each sample, most of the mineralized cells exhibit a lower ^13^C^14^N/^12^C^14^N ratio than non-mineralized cells. Mean ^13^C^14^N/^12^C^14^N ratios have been calculated and compared for each subpopulation (mineralized vs. non-mineralized cells) of each sample (**Figure 7**). After 4 days of biomineralization (in Lp, FeP, or Gt media) followed by 4 days in the labeled medium, mineralized cells from a given sample incorporated significantly less acetate than non-mineralized cells from the same sample (**Figure 7A**). Noteworthy, mineralized cells exhibited comparable ^13^C^14^N/^12^C^14^N distributions, whatever the composition of the biomineralization medium they came from.

In addition, we investigated the effect of the duration of biomineralization (t_min_) on carbon assimilation by mineralized cells. For that purpose, we collected samples at successive stages (4 h, 1 day, or 4 days) from the Gt-medium that we subsequently cultured in the labeled medium for 4 days (**Figure 7B**). ^13^C^14^N/^12^C^14^N ratios of mineralized cells decreased with increasing the biomineralization time. Interestingly, in samples only exposed for 4 h or 1 day to biomineralization conditions, carbon incorporation by mineralized and non-mineralized cells was rather similar (*p* = 9.2 10^-3^ and 3.1 10^-2^). In contrast, a much more significant lack of carbon incorporation by mineralized cells was observed for t_min_ = 4 days (*p* = 1.3 10^-16^).

In addition, we plotted ^13^C^14^N/^12^C^14^N ratios as a function of ^16^O/CN ratios for all the mineralized cells from all the samples (i.e., independently of the biomineralization conditions and time spent in the labeled medium; **Figure 4E**). These data could be fitted with a decreasing exponential curve. Noteworthy many mineralized cells exhibited a low ^16^O/CN ratio (but above the threshold, i.e., cells at early stages of biomineralization) and a high ^13^C^14^N/^12^C^14^N ratio (see e.g., red, green, and yellow points in **Figure 4E**, corresponding to bacteria circled in **Figures 4C,D**).

### Carbon Assimilation by Non-Mineralized Cells

Carbon assimilation has been followed in the non-mineralized subpopulations of each sample (black dots in **Figure 6**). Whatever the biomineralization conditions, ^13^C^14^N/^12^C^14^N ratio varied in the (0; 0.42) range, with an increase both of the mean and variance with increasing time in the labeled medium. It should be noted that the maximum was obtained for a culture grown for 4 h in the Gt-medium, then transferred for 4 days in the labeled medium (not depicted in **Figure 6**). We compared the distributions of ^13^C^14^N/^12^C^14^N ratios in the non-mineralized populations of samples mineralized for 4 days (in Lp, Mt, FeP, or Gt media) then cultured for 4 days in the labeled medium (**Figure 7A**). All these samples exhibited comparable ^13^C^14^N/^12^C^14^N distributions. Similarly, we followed ^13^C incorporation by the non-mineralized sub-population of the Gt sample as a function of time spent in the labeled medium (**Figure 7B**). Whereas C assimilation decreased with time in the mineralized cells, C incorporation by non-mineralized cells tended to remain rather constant with time.

## Discussion

### Periplasmic Encrustation Control over Carbon Assimilation and Viability

A diversity of nitrate reducing bacteria have been shown to promote Fe(II) oxidation and precipitation of Fe minerals (Kappler et al., 2005; Chakraborty et al., 2011; Carlson et al., 2012; Chakraborty and Picardal, 2013; Etique et al., 2014; Klueglein et al., 2014). Under biomineralization conditions, all these strains become progressively encrusted with Fe phases. In *Acidovorax* sp. strain BoFeN1, mineralization starts in the periplasm at the contact of the inner membrane then progressively encrusts the periplasmic space. Further, minerals deposit at the cell surface (Miot et al., 2011). At ultimate stages, minerals fill in the cytoplasm (Li et al., 2013a; Klueglein et al., 2014). In addition, existence of mineralized cells in division has been reported (Miot et al., 2011). However, it was unclear whether mineralized cells were still able to divide or whether mineralization occurred very rapidly during division hence freezing the cell division process. This raises the question of the chronology of Fe mineralization vs. cell death.

Here, we observed that carbon incorporation depended on the level of cell mineralization (**Figures 4** and **6**). At low cell encrustation levels, carbon assimilation by mineralized cells was comparable to that of the non-mineralized cells (**Figure 7**). However, carbon assimilation decreased exponentially with increasing cell mineralization by Fe minerals (**Figure 4E**). Interestingly, carbon assimilation by mineralized cells was not dependent on the nature of Fe minerals precipitated in the cell wall, but only on the amount of Fe precipitated in the periplasm and at the direct cell contact. There is thus a biomineralization threshold above which carbon assimilation ceases, but below which carbon assimilation is not or only partially hampered. Whereas carbon assimilation by mineralized cells dropped with increasing the level of periplasmic encrustation, carbon assimilation by non-mineralized cells remained constant (**Figure 7B**). Present results therefore strongly support that periplasmic encrustation and cell death are temporarily decoupled, these two processes occurring successively through the following scenario: as the periplasm becomes slightly mineralized, carbon assimilation, and cell division are still possible (Miot et al., 2011). Then, further cell wall mineralization progressively limits carbon assimilation, until reaching high Fe mineralization levels that lead to cessation of metabolic activity, and potentially cell lysis. Hence, these results suggest that first stages of periplasmic Fe biomineralization occur while bacteria are alive and not *post mortem*. Ultimate stages of mineralization leading to cytoplasm encrustation (Li et al., 2013a; Klueglein et al., 2014) most probably induce membrane perforation and cell death. Carlson et al. (2013) reported faster rates of Fe(II) oxidation by lysates compared with intact cells. Abiotic oxidation reactions may thus promote cytoplasmic precipitation of Fe minerals.

Our present results shed light on the impact of Fe biomineralization on carbon assimilation. Although we did not investigate more specifically the link with cell death, we can propose that periplasmic encrustation may induce multiple processes ultimately leading to cell death. Carlson et al. (2013) demonstrated that the main response to Fe biomineralization conditions was a stress response with an up-regulation of metal eﬄux pumps and of proteins involved in the response to nitrosative/redox stress. Inactivation of proteins from respiratory complexes (e.g., nitrite reductase, NO reductase) might occur in biomineralized cells, consistently with the accumulation of nitrate reduction intermediates in cultures of *Acidovorax ebreus* under nitrate dependent Fe(II) oxidation conditions (Carlson et al., 2013). In addition, although there is no proteomic evidence for a major role played by these processes, we cannot exclude that Fe biomineralization would directly inactivate proteins involved in C assimilation pathways. Biomineralization conditions may also lead to overexpression of membrane proteins such as porins (Carlson et al., 2013), which would promote pore formation as a step toward cell death (e.g., Bednarska et al., 2013). Eventually, periplasmic biomineralization would promote protein aggregation. Indeed, protein aggregates entrapped within the mineralized cell wall of BoFeN1 cells have been previously identified (Miot et al., 2011), and such protein aggregation has been associated with loss of function (Bednarska et al., 2013). Segregation of protein aggregates toward the old poles of bacterial cells is a common process limiting toxicity of protein aggregates and preserving cell integrity (e.g., Dougan et al., 2002). This process is consistent with previous observations of periplasmic biomineralization occurring preferentially at the old poles of the cells, while new septae remain free of Fe precipitates in BoFeN1 (Miot et al., 2011). At advanced stages of biomineralization, protein aggregation combined with mineral accumulation within the periplasm may induce stress response including reduction of membrane permeability (Ami et al., 2009). In the end, all the aforementioned processes would contribute to loss of viability.

### Link between Individual Phenotypic Variability of Carbon Assimilation and Fe Biomineralization

Our study evidences the co-existence of mineralized and non-mineralized cells at each stage of biomineralization and whatever the nature of Fe biominerals formed. Whereas mineralized cells did not incorporate carbon, moderately mineralized and non-mineralized cells significantly incorporated acetate. Previous studies evidenced persistence of acetate incorporation by some nitrate-reducing Fe(II) oxidizing strains even at advanced stages of Fe biomineralization (Klueglein et al., 2014). This has been observed not only for *Acidovorax* sp. strain BoFeN1 but also for *Pseudogulbenkania* sp. strain 2002 and for heterotrophic nitrate reducers such as *Paracoccus denitrificans* ATCC 19367 and *P. denitrificans* Pd 1222 (Klueglein et al., 2014). Our results are consistent with these previous observations, as acetate incorporation at advanced stages of Fe biomineralization can be attributed to metabolic activity of non-mineralized and moderately mineralized cells. Interestingly, low proportions of metabolically active cells (∼10%) were enough for the cultures to recover (**Figure 5**). This is consistent with previous estimations and suggestions that low proportions of non-mineralized cells could account for acetate consumption at advanced biomineralization stages (Klueglein et al., 2014).

Phenotypic variations within a monospecific bacterial culture under controlled conditions have been reported in multiple systems (e.g., Davidson and Surette, 2008; Raj and van Oudenaarden, 2008; Ackermann, 2013), shedding light on the fact that a microbial population is a heterogeneous group of physiologically distinct individuals. Individual variations are increasingly studied with the advent of powerful tools such as NanoSIMS allowing the exploration of metabolic processes down to the single cell level in microbial cultures and environmental samples (Musat et al., 2008, 2012; Dekas et al., 2009; Morono et al., 2011; Milucka et al., 2012; Remusat et al., 2012; Kopp et al., 2013; Zimmermann et al., 2015). Individual non-genetic phenotypic heterogeneity has been investigated for multiple traits including metabolism (e.g., Ackermann et al., 2008; Kiviet et al., 2014) and stress response (Balaban et al., 2004; Wakamoto et al., 2013; Holland et al., 2014). In particular, a subpopulation of bacteria known as “persisters” usually overcomes lethal stress induced by antibiotics, being responsible for antibiotic resistance (e.g., Lewis, 2010). It has been recently shown that stochastic expression of genes (non-responsive, selection-mediated adaptation) is a robust (in terms of fitness) alternative adaptation to stresses compared to the expression of dedicated repair mechanisms (sense-and-respond adaptative strategy; Wakamoto et al., 2013).

In the present study phenotypic cell-to-cell variability of carbon assimilation associated with individual variability of periplasmic Fe biomineralization may mirror non-genetic heterogeneity caused by different gene expression by individual bacteria. Although such a hypothesis would deserve a dedicated study, we may tentatively attribute these observed phenotypic differences to several individual differences in gene expression along the course of the cultures. Heterogeneities of Fe biomineralization may mirror (1) individual differences in response to the toxicity of Fe^2+^ and reactive nitrogen species, via the differential expression (up/down regulation) of metal eﬄux pumps and proteins involved in the cytoplasmic response to nitrosative stress (Carlson et al., 2013) and (2) variations in the individual rates of enzymatic nitrogen species reduction (NO_3_^-^, NO_2_^-^, NO, N_2_O; Carlson et al., 2013), themselves dependent on concentrations of organic co-substrates and access to nutrients (acetate, nitrate). (3) Finally, *Acidovorax* sp. strain BoFeN1 has been shown to produce EPS that get progressively mineralized with Fe minerals (Miot et al., 2009b). This has been observed in other nitrate-dependent Fe(II)-oxidizers as well, though with notable interspecific differences (Klueglein et al., 2014). Different individual rates of EPS production might partly account for different individual capabilities to overcome encrustation. This assumption might be tested in the future by correlating individual rates of carbon assimilation and Fe encrustation with local amounts of EPS produced, e.g., through the use of correlative NanoSIMS and carbon K-edge STXM analyses (Remusat et al., 2012).

### Phenotypic Heterogeneity as a Strategy to Cope with Fe Biomineralization?

In the present study, we analyzed bacteria from batch cultures exposed to relatively high (millimolar) Fe(II) concentrations. In contrast, in continuous flow systems at much lower Fe^2+^ concentrations, nitrate reducing Fe(II)-oxidizing bacteria were shown to exhibit minimized cell encrustation by Fe minerals (Chakraborty et al., 2011). Under these conditions, it was shown that nutrient uptake by *Acidovorax* sp. strain 2AN continued, whereas it ceased after 3–4 days of exposure to millimolar Fe^2+^ concentrations under batch conditions. Our results are consistent with these previous observations, showing that low levels of periplasmic encrustation do not significantly hamper carbon assimilation, hence suggesting that under low Fe^2+^ concentrations, Fe biomineralization would not significantly interfere with cell growth.

Besides, some modern and past environmental conditions might offer higher Fe(II) concentrations, comparable to those set in the present study. Indeed, high dissolved Fe^2+^ concentrations might have been predominant in the Precambrian ocean (Poulton and Canfield, 2011; Li et al., 2013b). Some modern environments exhibit millimolar Fe^2+^ concentrations as well. For instance, the deep anoxic layer of the meromictic lake Pavin displays Fe^2+^ concentrations at the saturation with the Fe phosphate vivianite (Viollier et al., 1997), comparable to the conditions reached in the Gt and FeP media used in the present study. Bacteria encrusted with Fe phosphate have been evidenced in this lake (Cosmidis et al., 2014), showing morphological and compositional similarities with encrusted nitrate reducing Fe(II)-oxidizers.

It is worth understanding why nitrate reducing Fe(II) oxidizing bacteria did not evolve pathways to avoid cell encrustation. Indeed, many bacterial groups have developed strategies to cope with metals, in particular Fe. On the one hand, some bacteria produce organics that template Fe mineral precipitation, thus avoiding cell encrustation. For instance, microaerobic Fe(II)-oxidizers such as *Gallionella* sp. and *Mariprofundus* sp., or *Leptothrix* sp. produce stalks or microtubes composed of organic fibers at the surface of which Fe minerals nucleate (Chan et al., 2011; Comolli et al., 2011; Saini and Chan, 2013). Extrusion of these organo-mineral structures leaves cells free of precipitates. On the other hand, phototrophic Fe(II)-oxidizers have been proposed to avoid encrustation by maintaining a local acidic pH in their surroundings (Hegler et al., 2010). Our results suggest that the periplasmic encrustation trait could have been maintained through (1) the limitation of periplasmic encrustation to low levels in environments exhibiting low Fe^2+^ concentrations, and (2) the persistence of a low proportion of cells remaining free of precipitates, while the majority of the cells do not avoid cell encrustation in more ferruginous environments. Further investigations would be needed to explore the underlying mechanisms and clarify how such bacteria may have persisted in natural ferruginous habitats despite the deleterious effects of periplasmic encrustation.

Noteworthy, depletion of C assimilation concomitantly with Fe biomineralization was observed whatever the composition of Fe biomineralization media (e.g., with or without phosphate) and the nature of Fe biominerals precipitated. As a consequence, we would expect that this link between individual phenotypic heterogeneities of Fe biomineralization and C assimilation would be ubiquitous in natural ferruginous environments.

Using a combination of NanoSIMS and flow cell sorting, phenotypic heterogeneities of carbon and nitrogen assimilation have been recently evidenced among different bacterial populations in the meromictic lake Cadagno (Musat et al., 2008; Zimmermann et al., 2015), suggesting potential non-genetic phenotypic diversity in this natural habitat. Here, we propose that Fe biomineralization would influence such non-genetic phenotypic variability under ferruginous conditions. In addition, our study shows that Fe biomineralization would provide a signature of the metabolic status of the cells. Exploring the relationship between phenotypic heterogeneity and biomineralization processes in natural habitats would provide a step toward understanding ecosystem functioning and evolutionary adaptation to metal-rich niches.

## Author Contributions

JM designed the experiments, acquired, interpreted data. LR designed, acquired and interpreted NanoSIMS data, ED performed the statistical analyses of NanoSIMS data, AG acquired NanoSIMS data, SP performed SEM analyses and MP contributed to microbial cultures. All authors contributed to manuscript writing and revision.

## Conflict of Interest Statement

The authors declare that the research was conducted in the absence of any commercial or financial relationships that could be construed as a potential conflict of interest.
