# Supplementary material for: Fe biomineralization mirrors individual metabolic activity in a nitrate-dependent Fe(II)-oxidizer
Source: Front Microbiol. 2015 Sep 8;6:879. doi: 10.3389/fmicb.2015.00879 (PMC4562303; doi:10.3389/fmicb.2015.00879)
Supplement: Supplementary file 1 [file Table_1.DOCX]

| **Biomineralization conditions** | | **t_lab_** | | |
| --- | --- | --- | --- | --- |
| **Medium** | **t_min_** | 4 hours | 1 day | 4 days |
| Lp | 4 days | 140 (99,29%) | 29 (100%) | 106 (1,89%) |
| Mt | 4 days | 18 (100%) | 13 (100%) | 130 (3,85%) |
| FeP | 4 days | 167 (77,25%) | 120 (37,5%) | 143 (13,99%) |
| Gt | 4 hours | - | 15 (40%) | 271 (12,55%) |
|  | 1 day | - | 132 (37,88%) | 183 (12,57%) |
|  | 4 days | 95 (88,42%) | 141 (13,48%) | 161 (23,6%) |

**Table SI1.** BoFeN1 cell dataset: number of ROI and proportion of mineralized cells for each couple of biomineralization and labeling conditions.
